# Disparities among Black and Hispanic colorectal cancer patients: Findings from the California Cancer Registry

**DOI:** 10.1002/cam4.6653

**Published:** 2023-11-01

**Authors:** Joel Sanchez Mendez, Ruoxuan Wang, Lihua Liu, Juanjuan Zhang, Stephanie L. Schmit, Jane Figueiredo, Heinz‐Josef Lenz, Mariana C. Stern

**Affiliations:** ^1^ Department of Population and Public Health Sciences, Keck School of Medicine University of Southern California Los Angeles California USA; ^2^ Los Angeles Cancer Surveillance Program University of Southern California Los Angeles California USA; ^3^ Norris Comprehensive Cancer Center University of Southern California Los Angeles California USA; ^4^ Genomic Medicine Institute Cleveland Clinic Cleveland Ohio USA; ^5^ Population and Cancer Prevention Program Case Comprehensive Cancer Center Cleveland Ohio USA; ^6^ Department of Medicine, Samuel Oschin Comprehensive Cancer Institute Cedars Sinai Medical Center Los Angeles California USA; ^7^ Department of Medicine, Keck School of Medicine of USC University of Southern California Los Angeles California USA

**Keywords:** African American, colorectal cancer, disparities, Hispanic, Latino, registry

## Abstract

**Background:**

Colorectal cancer (CRC) is the third most common cancer in California and second among Hispanic/Latinx (H/L) males. Data from the California Cancer Registry were utilized to investigate the differential impact on CRC outcomes from demographic and clinical characteristics among non‐Hispanic white (NHW), non‐Hispanic Black (NHB), U.S. born (USB), and non‐U.S. born (NUSB) H/L patients diagnosed during 1995–2020.

**Methods:**

We identified 248,238 NHW, 28,433 NHB, and 62,747 H/L cases (32,402 NUSB and 30,345 USB). Disparities across groups were evaluated through case frequencies, odds ratios (OR) from logistic regression, and hazard ratios (HR) from Cox regression models. All statistical tests were two‐sided.

**Results:**

NHB patients showed a higher proportion of colon tumors (75.8%) than NHW (71.5%), whereas both NUSB (65.9%) and USB (66.9%) H/L cases had less (*p* < 0.001). In multivariate models, NUSB H/L cases were 15% more likely than NHW to have rectal cancer. Compared to NHW, NHB cases had the greatest proportion of Stage IV diagnoses (26.0%) and were more likely to die of CRC (multivariate HR = 1.12; 95% CI = 1.10–1.15). Instead, NUSB H/L patients were less likely to die of CRC (multivariate HR = 0.87; 95% CI = 0.85–0.89) whereas USB H/L did not differ from NHW.

**Conclusions:**

NHB and H/L cases have more adverse characteristics at diagnosis compared to NHW cases, with NHB cases being more likely to die from CRC. However, NUSB H/Ls cases showed better survival than NHW and US born H/L patients. These findings highlight the importance of considering nativity among H/L populations to understand cancer disparities.

## Abstract 2

Data from the California Cancer Registry were utilized to evaluate the impact on colorectal cancer outcomes from demographic and clinical characteristics among non‐Hispanic White, non‐Hispanic Black, U.S. born, and non‐U.S. born Hispanic/Latino/a/x patients during 1995–2020. We report more adverse characteristics at diagnosis in non‐Hispanic Black and non‐hispanic White participants. Non‐U.S born Hispanic/Latino/x/a patients showed better survival relative to non‐Hispanic white and U.S. born patients.

## 1 INTRODUCTION

Colorectal cancer (CRC) is the third most common cancer in the USA, and the second cause of cancer death representing 8% of all new cancer cases.^1^ Non‐Hispanic Black (NHB) individuals have higher incidence of CRC compared to non‐Hispanic White (NHW) individuals and greater mortality.^2^ Among NHB populations CRC accounts for 14% and 11% of the cancer burden, and 10% and 9% of cancer mortality, among males and females, respectively.^2^

Incidence and mortality rates of CRC in the United States are lower among Hispanic individuals, also referred as Latino/a/x individuals (henceforth referred to as H/L individuals) than NHW individuals. However, cancer accounts for a higher proportion of the cancer burden among H/L populations than NHW, with 11% and 9% of deaths among men and women, respectively.^3^ Among US H/L individuals, CRC is the second and third most common cancer and most common cause of cancer death among males and females, respectively.^3^ Previously, we and others reported disparities in CRC incidence by region of origin, with H/L of Mexican origin having the lowest incidence.^4^, ^5^ Overall, the CRC patterns of occurrence, outcome disparities, and tumor characteristics among H/L individuals are less well characterized than among those who are NHW.^6^, ^7^, ^8^

In California, CRC is the fourth most common cancer and second cause of cancer death, with the highest incidence among NHB individuals, who represent ~5% of the California population. H/L individuals account for 39.3% of the California population and represent 25.9% of the total US H/L population.^9^ About 35% of H/L in California were born outside the mainland USA, with 82.4% coming from Mexico, 9.4% from Central America, 2.4% from South America, 0.7% from Cuba, 1.5% from Puerto Rico, and 3.6% from other origins.^9^

In this observational descriptive study, our main objective was to report key socio‐demographic, clinical characteristics, and survival patterns of CRC among H/Ls and NHB individuals living in California between 1995 and 2020, and compare them to those observed among NHW individuals, considering differences within H/L patients by nativity, given previous reports that suggest region of origin can be a source of heterogeneity in the H/L population.

## 2 METHODS

### 2.1 Case identification

We used data from the California Cancer Registry (CCR) including primary CRC cases who were at least 18 years old and diagnosed between 1995 and 2020. Cases were identified by National Cancer Institute (NCI) Surveillance, Epidemiology, and End Results Program (SEER) site codes (21041‐21052). H/L status was defined by the NAACCR Hispanic Identification Algorithm (NHIA).^10^ We assigned nativity as U.S. born (USB) and non‐U.S. born (NUSB) based on birthplace information. Individuals born in Puerto Rico were included among the NUSB H/L category, even though those born after 1941 are US born, given previously reported disparities between island and mainland born Puerto Rican individuals. For individuals with missing birthplace (42.37%), we used an algorithm previously reported to impute nativity,^11^ and had 3.18% of individuals with missing values. Combining nativity and race we created four mutually exclusive groups: NHW, NHB, USB H/L, and NUSB H/L. Cause of death were identified based on the International Classification of Diseases, 9th Revision and 10th Revision (ICD‐9 and ICD‐10).

### 2.2 Study variables

We considered the following sociodemographic variables: age at diagnosis (<62, 62–76, >76, based on tertiles of study cases), insurance status (not insured, managed care, Medicaid, Medicare, other), marital status (single, married, separated/divorced/widowed, unknown), religion (Roman catholic, None/agnostic/atheist, Christian not catholic, Jewish, other western, Eastern, unknown), nativity (non‐U.S. born, U.S. born), and neighborhood Socioeconomic Status (nSES) with quintiles as cut‐off point, and as a three‐level ordinal variable, which was constructed based on CCR's previously published area‐based methodologies,^12^, ^13^ grouping variables as: (1) Low: includes lower and lower‐middle nSES; (2) Middle nSES; (3) High: includes upper‐middle and highest nSES.

We considered the following clinical characteristics: cancer stage based on AJCC staging system (Stage I–IV), as well as the SEER summary stage at the time of diagnosis (localized, regional, distant), tumor size (<2, 2–5, and >5 cm), presence of lymph nodes involvement (N0 and N1), presence of metastasis (M0 and M1), and tumor location (colon vs. rectum, ICD‐O‐3 codes for colon are C180‐189 and C260, and ICD‐O‐3 codes for rectum are C199 and C209). The tumor location for the colon was further stratified into left‐sided (C185‐187), right‐sided (C180‐184), and not otherwise specified (NOS) (C188‐189 and C260). We considered the following treatment variables: surgery, chemotherapy, radiation, immunotherapy, hormone therapy, transplant, and other therapy. We also created a composite overall treatment status variable (no treatment, had treatment, treatment recommended or not given or refused), and we considered time from diagnosis to first line of treatment.

### 2.3 Statistical analyses

Comparison of case frequencies for all variables across racial/ethnic groups and comparisons within H/Ls by nativity, were done using Chi‐squared tests. Odds ratios (OR) and 95% confidence intervals (CIs) from logistic regression models were used to identify determinants of CRC tumor location, receipt of treatment, and time from diagnosis to treatment, considering key confounders as described in the appropriate tables. Hazard ratios (HR) and associated 95% CIs were estimated using multivariable Cox proportional hazards regression to evaluate survival differences between NHB and H/L relative to NHW. We tested the proportional hazard assumptions graphically by plotting log follow‐up time against log survival (“log–log” plot) and the survival curve derived from the Cox regression model (that imposes a proportional hazard assumption) against the Kaplan–Meier survival curve (without such assumption). Confounding analyses were conducted to determine covariate inclusion in multivariable models, a threshold of >10% in the ORs or HRs was a priori determined and implemented. Cross‐product interaction terms were fitted to evaluate potential effect modification by year of diagnosis and religion when appropriate, and multicollinearity was assessed with variance inflation factor (VIF) computation with values of VIF <10 at individual covariate‐strata deemed acceptable. All analyses were done using STATA software, version 15.1 (StataCorp LLC, Texas). All tests of statistical significance are two‐sided. A *p*‐value <0.05 was deemed statistically significant.

## 3 RESULTS

Between 1995 and 2019, there were 343,867 CRC cases diagnosed in California among NHW (248,238), NHB (28,433), and H/L (62,747) patients, with 4449 individuals having missing race information. Among H/L, 30,345 (48.4%) were USB H/L and 32,402 (51.6%) were NUSB H/L patients.

### 3.1 Sociodemographic and clinical characteristics of CRC cases in California

Statistically significant differences were observed for year and age at diagnosis, nSES, insurance status, marital status, sex, and religion between NHB, USB H/L, and NUSB H/L cases, when compared to NHW cases (*p*‐values <0.001), and between USB H/L and NUSB H/L cases (*p*‐values <0.001) (Table 1). We observed a decrease in the proportion of NHWs diagnosed from 2005 onwards, with an increase in the percentage of NHB, USB H/Ls and NUSB H/Ls in more recent years. Moreover, a greater proportion of diagnoses before age 62 among USB H/L (45.6%) and NUSB H/L patients (42.1%) compared to NHB (37.4%) and NHW (26.6%) cases was found. Compared to NHW patients, USB H/L, and NUSB H/L patients had more than double the proportion of diagnoses younger than 50 years old (7.7% NHW vs. 18.2% USB H/Ls and 15.1% NUSB H/Ls, 11% NHB) and were on average, of lower nSES, uninsured, married, and Roman Catholic. Across all racial/ethnic categories there was higher proportion of diagnoses among females than males, with the highest proportion among USB H/L patients (55.4%), followed by NUSB H/L patients (53.2%).

**TABLE 1:** Sociodemographic characteristics of NHW, NHB, and H/L CRC patients in California (1995–2020).

	NHW	NHB	USB H/Ls	NUSB H/Ls	Heterogeneity
	*N* = 248,238	*N* = 28,433	*N* = 30,345	*N* = 32,402	*p*‐value
Year of diagnosis					<0.001
1995–2004	108,158 (43.6%)	10,736 (37.8%)	8930 (29.4%)	8640 (26.7%)	
2005–2014	93,033 (37.5%)	11,850 (41.7%)	12,798 (42.2%)	13,911 (42.9%)	
2015 onwards	47,047 (19.0%)	5847 (20.6%)	8617 (28.4%)	9851 (30.4%)	
*p*‐value*		<0.001	<0.001	<0.001	
*p*‐value**				<0.001	
Age at diagnosis					<0.001
<62	66,020 (26.6%)	10,636 (37.4%)	13,835 (45.6%)	13,636 (42.1%)	
62–76	92,735 (37.4%)	11,279 (39.7%)	11,019 (36.3%)	11,376 (35.1%)	
>76	89,483 (36.0%)	6518 (22.9%)	5491 (18.1%)	7390 (22.8%)	
*p*‐value*		<0.001	<0.001	<0.001	
*p*‐value **				<0.001	
Sex					
Females	128,502 (51.8%)	14,237 (50.1%)	16,821 (55.4%)	17,229 (53.2%)	
Males	119,697 (48.2%)	14,193 (49.9%)	13,517 (44.6%)	15,165 (46.8%)	
*p*‐value*		<0.001	<0.001	<0.001	
*p*‐value**				<0.001	
nSES					<0.001
Low nSES	67,805 (27.3%)	15,699 (55.2%)	15,812 (52.1%)	20,424 (63.0%)	
Middle nSES	56,014 (22.6%)	5696 (20.0%)	6577 (21.7%)	5633 (17.4%)	
High nSES	124,419 (50.1%)	7038 (24.8%)	7956 (26.2%)	6345 (19.6%)	
*p*‐value*		<0.001	<0.001	<0.001	
*p*‐value**				<0.001	
Insurance status					<0.001
Not insured	2400 (1.0%)	564 (2.0%)	513 (1.7%)	1278 (3.9%)	
Managed care	109,168 (44.0%)	12,233 (43.0%)	14,989 (49.4%)	11,838 (36.5%)	
Medicaid	9320 (3.8%)	2748 (9.7%)	3023 (10.0%)	5937 (18.3%)	
Medicare	105,637 (42.6%)	9870 (34.7%)	9318 (30.7%)	10,885 (33.6%)	
Other	7194 (2.9%)	1438 (5.1%)	1110 (3.7%)	771 (2.4%)	
Unknown	14,519 (5.8%)	1580 (5.6%)	1392 (4.6%)	1693 (5.2%)	
*p*‐value*		<0.001	<0.001	<0.001	
*p*‐value **				<0.001	
Marital status					<0.001
Single	32,596 (13.1%)	7349 (25.8%)	5840 (19.2%)	5327 (16.4%)	
Married	131,643 (53.0%)	10,892 (38.3%)	15,988 (52.7%)	18,363 (56.7%)	
Separated/divorced/widowed	74,180 (29.9%)	8669 (30.5%)	7183 (23.7%)	7237 (22.3%)	
Unknown	9819 (4.0%)	1523 (5.4%)	1334 (4.4%)	1475 (4.6%)	
*p*‐value*		<0.001	<0.001	<0.001	
*p*‐value**				<0.001	
Religion					<0.001
Roman Catholic	37,967 (15.3%)	1540 (5.4%)	14,401 (47.5%)	18,864 (58.2%)	
None/agnostic/atheist	17,919 (7.2%)	1280 (4.5%)	1260 (4.2%)	936 (2.9%)	
Christian not Catholic	80,953 (32.6%)	14,194 (49.9%)	5391 (17.8%)	3981 (12.3%)	
Jewish	8274 (3.3%)	49 (0.2%)	71 (0.2%)	85 (0.3%)	
Other Western	261 (0.1%)	30 (0.1%)	11 (<1%)	12 (<1%)	
Eastern	1355 (0.6%)	163 (0.6%)	28 (0.1%)	24 (0.1%)	
Unknown	101,509 (40.9%)	11,177 (39.3%)	9183 (30.3%)	8500 (26.23%)	
*p*‐value*		<0.001	<0.001	<0.001	
*p*‐value**				<0.001	

Abbreviations: CRC, Colorectal Cancer; NHB, Non‐Hispanic Blacks; NHW, Non‐Hispanic Whites; nSES, Neighborhood‐socioeconomic status; NUSB H/L, non‐US born Hispanic/Latino/x/a; USB H/L, US born Hispanic/Latino/x/a.

* Versus NHW.

** USB H/L versus NUSB H/L patients.

Disparities in clinical characteristics were found (Table 2). NHB cases tended to have a higher proportion of colon tumors than NHW patients (74.6% in NHB vs. 71.5% in NHW), with 43.3% of those being right‐sided among NHB cases. In contrast, USB H/L patients showed a higher proportion of rectal tumors when compared to NHW patients (33.1% vs. 28.5%, respectively) which increased among NUSB H/L cases (34.1%) when compared to USB H/L patients. A greater proportion of more adverse tumor characteristics were observed among NHB and USB H/L patients when compared to NHW patients. For example, a greater proportion of larger tumors (5+ cm), more advanced AJCC stage, distant infiltration, metastasis, and prolonged time to treatment reception (3–6 months) were observed at diagnosis (all *p*‐values <0.001).

**TABLE 2:** Clinical characteristics of NHW, NHB, and H/L CRC patients in California (1995–2020).

(All percentages are calculated among those with available data only)	NHW	NHB	USB H/Ls	NUSB H/Ls	Heterogeneity
	*N* = 248,238	*N* = 28,433	*N* = 30,345	*N* = 32,402	*p*‐value
Tumor location					<0.001
Colon	174,243 (71.5%)	21,200 (75.8%)	19,843 (66.9%)	20,911 (65.9%)	
Rectum	69,387 (28.5%)	6778 (24.2%)	9813 (33.1%)	10,838 (34.1%)	
Missing	4608	455	689	653	
*p*‐value*		<0.001	<0.001	<0.001	
*p*‐value**				0.006	
Tumor laterality					<0.001
Right colon	98,798 (40.6%)	12,113 (43.3%)	10,193 (34.4%)	10,911 (34.4%)	
Left colon	64,470 (26.5%)	7727 (27.6%)	8482 (28.6%)	8574 (27.0%)	
Colon NOS	10,975 (4.5%)	1360 (4.9%)	1168 (3.9%)	1426 (4.5%)	
Rectum	69,387 (28.5%)	6778 (24.2%)	9813 (33.1%)	10,838 (34.1%)	
Missing	4608	455	689	653	
*p*‐value*		<0.001	<0.001	<0.001	
*p*‐value**				<0.001	
Tumor size					<0.001
<2 cm	25,328 (13.8%)	3002 (15%)	3385 (15%)	3196 (13.5%)	
2–5 cm	99,485 (54.2%)	10,345 (51.5%)	11,356 (50.4%)	11,687 (49.4%)	
5+ cm	58,703 (32.0%)	6727 (33.5%)	7813 (34.6%)	8791 (37.1%)	
Missing	64,722	8359	7791	8728	
*p*‐value*		<0.001	<0.001	<0.001	
*p*‐value**				<0.001	
AJCC Stage					<0.001
Stage I	55,106 (26.5%)	5454 (23.9%)	6219 (24.6%)	5908 (22.1%)	
Stage II	58,802 (28.2%)	5588 (24.5%)	6470 (25.5%)	7245 (27.1%)	
Stage III	52,273 (25.1%)	5826 (25.6%)	6876 (27.2%)	7344 (27.5%)	
Stage IV	42,045 (20.2%)	5914 (26.0%)	5760 (22.7%)	6213 (23.3%)	
Missing	40,012	5651	5020	5692	
*p*‐value*		<0.001	<0.001	<0.001	
*p*‐value**				<0.001	
Summary stage at time of diagnosis					<0.001
Localized	92,018 (41.3%)	9798 (39.2%)	10,768 (39.1%)	10,917 (37.4%)	
Regional	84,894 (38.1%)	8845 (35.4%)	10,447 (38.0%)	11,489 (39.4%)	
Distant	45,737 (20.5%)	6329 (25.3%)	6305 (22.9%)	6745 (23.1%)	
Missing	25,589	3461	2825	3251	
*p*‐value*		<0.001	<0.001	<0.001	
*p*‐value**				<0.001	
Lymph nodes					<0.001
N0	67,018 (87.7%)	8239 (85.8%)	9017 (87.2%)	9491 (87.5%)	
N1	9392 (12.3%)	1365 (14.2%)	1321 (12.8%)	1357 (12.5%)	
Missing	171,828	18,829	20,007	21,554	
*p*‐value*		<0.001	0.158	0.519	
*p*‐value**				0.556	
Metastasis					<0.001
M0	91,896 (82.5%)	11,093 (79.0%)	12,512 (81.5%)	13,522 (80.7%)	
M1	19,447 (17.5%)	2953 (21.0%)	2834 (18.5%)	3224 (19.3%)	
Missing	136,895	14,387	14,999	15,656	
*p*‐value*		<0.001	0.002	<0.001	
*p*‐value**				0.073	
Treatment status					<0.001
No treatment recorded	16,207 (6.6%)	2358 (8.4%)	1757 (5.9%)	2278 (7.1%)	
Had treatment	220,659 (90.3%)	24,458 (87.5%)	27,320 (91.2%)	28,524 (89.5%)	
Treatment recommended but not given/refused	7467 (3.1%)	1147 (4.1%)	864 (2.9%)	1086 (3.4%)	
Missing	3905	470	404	514	
*p*‐value*		<0.001	<0.001	<0.001	
*p*‐value**				<0.001	
Treatment type					<0.001
None	23,674 (9.7%)	3505 (12.5%)	2621 (8.8%)	3364 (10.5%)	
Radiation	4799 (2.0%)	596 (2.1%)	691 (2.3%)	863 (2.7%)	
Surgery	143,610 (58.8%)	15,567 (55.7%)	15,597 (52.1%)	16,055 (50.3%)	
Other	72,250 (29.6%)	8295 (29.7%)	11,032 (36.8%)	11,606 (36.4%)	
Missing	3905	470	404	514	
*p*‐value*		<0.001	<0.001	<0.001	
*p*‐value**				<0.001	
Time from diagnosis to treatment					<0.001
No treatment	23,674 (9.8%)	3505 (12.6%)	2621 (8.8%)	3364 (10.6%)	
0–3 months	196,552 (81.0%)	21,474 (77.2%)	23,981 (80.6%)	24,551 (77.6%)	
3–6 months	3589 (1.5%)	740 (2.7%)	711 (2.4%)	1021 (3.2%)	
6–12 months	711 (0.3%)	154 (0.6%)	146 (0.5%)	191 (0.6%)	
12+ months	18,001 (7.4%)	1930 (6.9%)	2288 (7.7%)	2498 (7.9%)	
Missing	5711	630	598	777	
*p*‐value*		<0.001	<0.001	<0.001	
*p*‐value**				<0.001	

Abbreviations: CRC, Colorectal Cancer; NHB, Non‐Hispanic Blacks; NHW, Non‐Hispanic Whites; NOS, Not otherwise specified; NUSB H/L, non‐US born Hispanic/Latino/x/a; USB H/L, US born Hispanic/Latino/x/a.

* Versus NHW.

** USB H/L versus NUSB H/L patients.

When compared to USB H/L, NUSB H/L patients were less likely to have tumors <2 cm, with a smaller proportion of Stage I diagnosis, localized lesions, and receipt of treatment, including surgery (all *p*‐values <0.001). No statistically significant difference were reported for lymph node involvement and metastasis (*p*‐values = 0.556 & 0.073, respectively) (Table 2).

### 3.2 Determinants of tumor localization, treatment receipt, and time to treatment reception

When contrasting patients with rectum versus colon tumor localization, sex, age, and marital status were statistically significant determinants using multivariable analyses (Table 3). Compared to NHW cases, NHB patients were 29% less likely to have cancer in the rectum (OR = 0.71, 95% CI = 0.70–0.74, *p* < 0.001), whereas USB H/L cases were 6% (OR = 1.06, 95% CI = 1.03–1.08, *p* < 0.001) and NUSB H/L cases were 15% (OR = 1.15, 95% CI = 1.12–1.18, *p* < 0.001) more likely to have a tumor in the rectum than in the colon (Table 3). Being older, and being female were associated with reduced risk of rectal tumor localization (Table 3). Married cases were slightly less likely to have rectal tumors than single cases.

**TABLE 3:** Multivariable logistic regression models for determinants of tumor localization, receipt of treatment and time between diagnosis and treatment in California CRC patients (1995–2020).

	Tumor localization		*p*‐value	Receipt of treatment		*p*‐value	Time to treatment		*p*‐value
	Colon	Rectum		No treatment	Treatment received		0–3 months	3+ months	
	*N* = 238,832	*N* = 98,423		*N* = 33,933	*N* = 304,563		*N* = 269,686	*N* = 32,430	
	Odds ratio (95% CI)			Odds ratio (95% CI)			Odds ratio (95% CI)		
Population									
NHW	1^ref^			1^ref^			1^ref^		
NHB	0.71 (0.70–0.74)		<0.001	0.73 (0.69–0.78)		<0.001	1.14 (1.09–1.19)		<0.001
USB H/L	1.06 (1.03–1.08)		0.002	0.92 (0.86–0.89)		0.013	1.14 (1.09–1.19)		<0.001
NUSB H/L	1.15 (1.12–1.18)		<0.001	0.87 (0.81–0.92)		<0.001	1.27 (1.22–1.32)		<0.001
Age at diagnosis									
<62	1^ref^			1^ref^			1^ref^		
62–76	0.64 (0.63–0.65)		<0.001	0.51 (0.48–0.53)		<0.001	1.10 (1.07–1.14)		<0.001
>76	0.43 (0.42–0.44)		<0.001	0.19 (0.18–0.20)		<0.001	1.09 (1.05–1.14)		<0.001
Sex									
Male	1^ref^			1^ref^			1^ref^		
Female	0.79 (0.78–0.81)		<0.001	1.04 (1.01–1.08)		0.021	0.95 (0.93–0.98)		<0.001
Marital status									
Single	1^ref^			1^ref^			1^ref^		
Married	0.96 (0.94–0.98)		0.001	1.79 (1.70–1.88)		<0.001	0.93 (0.89–0.96)		<0.000
Separated/divorced/widow	0.98 (0.96–1.01)		0.162	1.18 (1.12–1.24)		<0.001	0.96 (0.92–1.00)		0.054
Unknown	1.20 (1.15–1.24)		<0.001	0.78 (0.71–0.85)		<0.001	1.07 (0.99–1.15)		0.101
nSES									
Low nSES	–			1^ref^		<0.001*	1^ref^		<0.001*
Middle nSES	–			1.14 (1.09–1.20)		<0.001	0.99 (0.96–1.03)		0.676
High nSES	–			1.33 (1.28–1.39)		<0.001	0.94 (0.91–0.97)		<0.001
Insurance status									
Not insured	–			1^ref^			1^ref^		
Managed care	–			2.83 (2.52–3.18)		<0.001	1.00 (0.90–1.12)		0.973
Medicaid	–			1.68 (1.48–1.90)		<0.001	1.25 (1.11–1.40)		<0.000
Medicare	–			2.43 (2.16–2.74)		<0.001	1.03 (0.92–1.15)		0.629
Other	–			1.96 (1.69–2.26)		<0.001	1.19 (1.05–1.35)		0.007
Unknown	–			1.50 (1.32–1.72)		<0.001	0.94 (0.83–1.07)		0.362
Religion									
Roman Catholic	–			1^ref^			–		
None/Agnostic/Atheist	–			0.77 (0.71–0.83)		<0.000	–		
Christian non‐Catholic	–			0.98 (0.93–1.04)		0.529	–		
Jewish	–			1.17 (1.03–1.32)		0.019	–		
Other Western	–			0.93 (0.51–1.70)		0.807	–		
Eastern	–			0.89 (0.68–1.15)		0.367	–		
Unknown	–			0.69 (0.70–0.72)		<0.000	–		
AJCC Stage									
Stage I	–			1^ref^			1^ref^		
Stage II	–			3.28 (3.07–3.51)		<0.001	1.12 (1.08–1.16)		<0.001
Stage III	–			6.75 (6.14–7.43)		<0.001	1.08 (1.05–1.12)		<0.001
Stage IV	–			0.16 (0.15–0.17)		<0.001	1.23 (1.18–1.27)		<0.001
Tumor location									
Colon	–			1^ref^			1^ref^		
Rectum	–			0.78 (0.75–0.81)		0.015	1.33 (1.29–1.36)		<0.001

Abbreviations: CRC, Colorectal Cancer; NHB, Non‐Hispanic Blacks; NHW, Non‐Hispanic Whites; nSES, neighborhood‐socioeconomic status; NUSB H/L, non‐US born Hispanic/Latino/x/a; USB H/L, US born Hispanic/Latino/x/a.

* *p*‐trend; five‐strata nSES variable (with quintiles as cut‐off point) was fitted as a continuous variable in independent model.

When colon tumors with laterality information were analyzed (*n* = 223,728), and after adjustment for age at diagnosis, sex, and marital status we observed that NHB patients were 0.85 times less‐likely to have left‐sided tumors when compared to NHW patients (95% CI: 0.83–0.88, *p* < 0.001), while USB H/L and NUSB H/L patients were 6% and 5% more likely to have left‐sided tumors (OR = 1.06, 95% CI: 1.03–1.10, *p* < 0.001 & OR = 1.05, 95% CI: 1.02–1.08, *p* < 0.001, respectively) (Table S1).

Comparing patients who did not receive treatment to those who did, and independent of age at diagnosis, sex, marital status, nSES, insurance status, religion, tumor stage, and location. We observed that NHB (OR = 0.73; 95% CI = 0.69–0.78, *p* < 0.001), USB (OR = 0.92; 95% CI = 0.86–0.98, *p* = 0.013), and NUSB H/L patients (OR = 0.87; 95% CI = 0.81–0.92, *p* < 0.001) were less likely than NHW to receive treatment (Table 3). No statistically significant interactions were reported between religion and race/ethnicity (*p*_interaction_ >0.05).

Among CRC cases who did not receive treatment, we observed that H/L patients had higher proportion of diagnoses from 2015 onwards (USB H/L 37%, NUSB H/Ls 35.4%, NHB 25.3%, and NHW 25.1%, *p* < 0.001) at a younger age (USB H/L 30.4% and NUSB H/L 26.4% vs. NHW 14.4%, *p* < 0.001) and male cases (USB H/Ls 59.3%, NUSB H/Ls 51.7%, NHB 47.9%, and NHW 48.4%, *p* < 0.001) when compared to NHW. Also, USB H/L and NUSB H/L cases (35.3% in both) had statistically significant higher proportions of rectal tumors than NHW cases (29.5%) (*p* < 0.001), with NHB patients having the greatest proportion of colon tumors (73.2%), 34.2% of those right‐sided (Table S2).

When determinants of receipt of first line of treatment within 3 months after CRC diagnosis were evaluated, we observed that NHB (OR = 1.14, 95% CI = 1.09–1.19), USB H/L (OR = 1.14, 95% CI = 1.09–1.19) and NUSB H/L (OR = 1.27, 95% CI = 1.22–1.32) patients were all more likely to receive treatment after 3 months since diagnosis compared to NHW patients, with the largest disparity for NUSB H/L cases, after adjustment for age at diagnosis, sex, marital status, nSES, insurance, AJCC stage and tumor location (Table 3). Cases with high nSES were more likely to receive treatment within 3 months compared to those with low nSES. Patients on Medicaid were more likely to be diagnosed after 3 months (OR = 1.25; 95% CI = 1.22–1.40) compared to those without insurance. Patients diagnosed with cancer in the rectum or with stage II‐IV were also more likely to receive treatment after 3 months of diagnosis. Compared to single cases, married CRC cases were less likely to have treatment after 3 months from date of diagnosis (Table 3).

### 3.3 Survival analysis

Results from multivariate analyses (Table 4) showed that when compared to NHW, NHB patients were 12% more likely to die of CRC (HR = 1.12; 95% CI = 1.10–1.15, *p* < 0.001), whereas NUSB H/L patients were less likely to die (HR = 0.87; 95% CI = 0.85–0.89, *p* < 0.001). No difference was reported between USB H/L and NHW cases (*p* = 0.68). Characteristics associated with higher risk of CRC death included being male, older age, low nSES, not receiving treatment, uninsured status, being single, rectal localization, and higher stage. When laterality was evaluated, CRC patients with left‐sided tumors were 7% less likely to die of CRC when compared to patients with right‐sided tumors (HR = 0.93, 95% CI: 0.91–0.95, *p* < 0.001). No interaction with year of diagnosis and race/ethnicity was reported (all *p*_interaction_ >0.05).

**TABLE 4:** Multivariate CRC‐specific survival analyses for CRC cases in California (1995–2020).

	Hazard ratio (95% CI)	*p*‐value
Population		
NHW	1^ref^	
NHB	1.12 (1.10–1.15)	<0.001
USB H/Ls	1.01 (0.98–1.03)	0.681
NUSB H/Ls	0.87 (0.85–0.89)	<0.001
Sex		
Males	1^ref^	
Females	0.93 (0.92–0.95)	<0.001
Age at diagnosis		
<62	1^ref^	
62–76	1.27 (1.25–1.30)	<0.001
>76	1.90 (1.86–1.94)	<0.001
nSES		
Low nSES	1^ref^	<0.001*
Middle nSES	0.96 (0.94–0.98)	<0.001
High nSES	0.88 (0.87–0.90)	<0.001
Marital status		
Single	1^ref^	
Married	0.86 (0.85–0.88)	<0.001
Separated/divorced/widowed	1.01 (0.99–1.04)	0.197
Unknown	0.91 (0.87–0.95)	<0.001
Insurance status		
Not insured	1^ref^	
Managed care	0.78 (0.73–0.82)	<0.001
Medicaid	0.93 (0.88–0.99)	0.019
Medicare	0.81 (0.77–0.86)	<0.001
Other	0.81 (0.76–0.87)	<0.001
Unknown	0.97 (0.91–1.03)	0.287
Tumor location		
Colon	1^ref^	
Rectum	1.15 (1.13–1.17)	<0.001
Treatment status		
No treatment	1^ref^	
Treatment received	0.33 (0.32–0.33)	<0.001
AJCC Stage		
Stage I	1^ref^	
Stage II	2.03 (2.0–2.1)	<0.001
Stage III	4.19 (4.1–4.3)	<0.001
Stage IV	20.4 (19.9–20.9)	<0.001

Abbreviations: NHB, Non‐Hispanic Blacks; NHW, Non‐Hispanic Whites; nSES, neighborhood‐socioeconomic status; NUSB H/L, non‐US born Hispanic/Latino/x/a; USB H/L, US‐born Hispanic/Latino/x/a.

* *p*‐trend; five‐strata nSES variable (with quintiles as cut‐off point) was fitted as a continuous variable in independent model.

Stage‐specific survival analyses suggested that compared to NHW individuals, the increased risk of death among NHB individuals was larger for Stage I and Stage II, and the reduced risk of CRC death among NUSB H/L individuals was larger among Stage IV patients (Table S3). Moreover, whereas analyses adjusting for stage showed that USB H/Ls individuals did not differ in risk of death compared to NHW individuals (Table 4), stratified analyses by stage at diagnosis showed that USB H/L patients had 11% higher risk of dying of CRC compared to NHW patients for stage II CRC (Table S3).

## 4 DISCUSSION

We report disparities regarding sociodemographic, clinical characteristics, and survival for CRC patients of different racial/ethnic groups in California, and among H/L patients when considering nativity. Key disparities included a greater proportion of younger diagnoses among USB H/L patients and rectal tumors among all H/L, when compared to NHW cases. In addition, we observed that NHB patients had more adverse characteristics at diagnosis, such as the greatest proportion of Stage IV cancer, and presence of metastases, followed by NUSB H/L patients. Importantly, compared to NHW, NHB and H/L patients were less likely to receive treatment and were more likely to receive it after 3 months from diagnosis. Consistent with these disparities, NHB patients were more likely to die of CRC compared to NHW cases. In contrast, despite being diagnosed with many adverse characteristics, USB H/L cases did not differ from NHW in their likelihood of dying of CRC from NHW, and NUSB H/L cases had lower likelihood of dying.

Our finding of higher risk of death due to CRC among NHB compared to NHW is consistent with previous reports using SEER‐wide data.^14^, ^15^, ^16^ A study using CCR data suggests the survival disparity between NHB and NHW years has disappeared starting in 2009; however, we saw no statistically significant interactions between year of diagnosis and race/ethnicity.^17^ Our finding of comparable risk of death for USB H/L and NHW, but reduced risk of death for NUSB H/L is supported by previous studies done in California,^11^, ^17^ both adjusting for similar covariates as we did in our study. In addition, previous SEER studies also reported no differences in survival or reduced risk of death for H/L patients compared to NWH, although neither study considered nativity,^14^, ^15^, ^16^ and one did not consider clinical variables.^16^ In addition, a study in Nevada also reported that H/L patients had improved survival compared to NHW, when considering various demographic factors, albeit not nativity, as well as stage and tumor characteristics.^18^

Studies focusing on mortality have either reported no differences in mortality for H/L patients when compared to NHW, without consideration of SES or nativity,^19^ or reduced mortality rates for H/L compared to NHW in California and Texas.^20^ Interestingly, in the latter study, when disaggregating by nativity, US born H/L males had higher mortality rates than NHW whereas NUSB H/L males and females had reduced mortality in both states. Female USB H/L patients in California also had reduced mortality compared to NHW, whereas those in Texas did not differ from NHW.^20^ Finally, a SEER‐based study also reported on lower mortality rates for H/L patients and reported that this mortality gap is narrowing, with NHW showing more than double the decline in mortality over the years 2000–2011 compared to H/L patients.^21^

In contrast to the above findings, 10% higher CRC‐related risk of dying was reported for H/L patients in a study with SEER data between 1988 and 2000, for H/L patients of Mexican origin and H/L patients of unknown origin, but not among those with origin in Puerto Rican, Cuban or South/Central America.^22^ This study considered treatment received but not SES or nativity. Another study in Texas also reported that H/L patients had ~16% increased risk of dying of CRC compared to NHW,^23^ however, this study did not consider treatment received or nativity. Finally, several single‐center studies across the US have reported that despite receiving similar treatments, H/L patients have worse survival than NHW.^24^, ^25^, ^26^ However, these studies did not consider differences by stage, nativity, SES, or treatment received,^24^, ^25^ or were focused only on metastatic cases and did not adjust for SES or nativity.^26^ In our study, we provide evidence that nSES is a confounder in the association between race/ethnicity, receipt of treatment, time to treatment, and CRC mortality. Furthermore, higher nSES was inversely correlated with increased likelihood of no treatment reception, prolonged time to diagnosis, and CRC‐specific mortality in a linear fashion (all *p*‐trend >0.005) (Tables 3 and 4).

Overall, the observed improved CRC‐specific survival among NUSB H/L patients compared to USB H/L and NHW patients is counter‐intuitive given the adverse clinical characteristics displayed in greater proportion among NUSB H/L patients. Whether this advantage is due to socio‐demographic characteristics, such as increased social support among NUSB H/L patients living in ethnic enclaves, or better health determinants (e.g., diet, lifestyle factors) as previously proposed,^27^ or biological differences given potential different genetic admixture patterns among NUSB H/L compared to USB H/L patients, is unclear. Finally, we cannot discard the possibility that some NUSB H/L individuals may have returned to their countries of origin, thus not being captured by vital statistics records in the US. Whether this “salmon hypothesis bias” may fully account for the observed survival advantage reported here and in other studies remains to be established.^27^, ^28^, ^29^ More studies are needed to understand the key determinants of this potential survival advantage. What is learned for the NUSB H/L population could potentially inform recommendations to improve survival among NHW, NHB, and USB H/L cancer patients.

Strengths of our study include the use of large data from the CCR, with large representation of H/L patients, and consideration of nativity which allowed us to uncover possible disparities within H/L individuals. The main weaknesses of our study are the missing values of cancer stage based on Stage SEER for approximate 15% of cases, non‐specified country of origin on 31.9% of NUSB H/Ls; which precluded analyses based on this variable, and missing nativity for 42.37% of study participants. However, sensitivity analysis showed that the distribution of sociodemographic and clinical variables was not affected by the imputation algorithm except in the dichotomous treatment reception variable, which showcased no statistically significant difference between NUSB H/Ls when compared to NHW and USB H/Ls (*p*‐value = 0.783 & 0.076, respectively) (Table S4). Furthermore, when multivariable models were fitted with non‐imputed nativity participants, all associations remained virtually unchanged when compared to main findings (Table S5).

In summary, our study reports disparities in CRC survival as well as sociodemographic and clinical characteristics among H/L and NHB patients compared with NHW in California. These findings highlight the importance of considering nativity in the evaluation of cancer patterns among H/L populations and the need to conduct follow‐up studies to understand the possible determinants captured by nativity that may explain the observed differences in survival.

## AUTHOR CONTRIBUTIONS

**Joel Sanchez Mendez:** Data curation (equal); formal analysis (equal); investigation (equal); methodology (equal); project administration (equal); visualization (equal); writing – original draft (equal); writing – review and editing (equal). **Ruoxuan Wang:** Conceptualization (equal); formal analysis (equal); investigation (equal); methodology (equal); supervision (equal); visualization (equal); writing – original draft (equal); writing – review and editing (equal). **Lihua Liu:** Formal analysis (equal); investigation (equal); methodology (equal); project administration (equal); validation (equal); visualization (equal); writing – original draft (equal); writing – review and editing (equal). **Juanjuan Zhang:** Conceptualization (equal); investigation (equal); methodology (equal); project administration (equal); validation (equal); visualization (equal); writing – original draft (equal); writing – review and editing (equal). **Stephanie L. Schmit:** Conceptualization (equal); data curation (equal); investigation (equal); methodology (equal); project administration (equal); software (equal); supervision (equal); validation (equal); writing – original draft (equal); writing – review and editing (equal). **Jane Figueiredo:** Funding acquisition (equal); investigation (equal); methodology (equal); project administration (equal); resources (equal); validation (equal); visualization (equal); writing – original draft (equal); writing – review and editing (equal). **Heinz‐Josef Lenz:** Conceptualization (equal); investigation (equal); methodology (equal); validation (equal); visualization (equal); writing – original draft (equal); writing – review and editing (equal). **Mariana C. Stern:** Conceptualization (equal); data curation (equal); formal analysis (equal); funding acquisition (equal); investigation (equal); methodology (equal); project administration (equal); resources (equal); supervision (equal); validation (equal); visualization (equal); writing – original draft (lead); writing – review and editing (lead).

## FUNDING INFORMATION

The collection of cancer incidence data used in this study was supported by the California Department of Public Health pursuant to California Health and Safety Code Section 103885; Centers for Disease Control and Prevention's (CDC) National Program of Cancer Registries, under cooperative agreement 5NU58DP006344; the National Cancer Institute's Surveillance, Epidemiology and End Results Program under contract HHSN261201800032I awarded to the University of California, San Francisco, contract HHSN261201800015I awarded to the University of Southern California, and contract HHSN261201800009I awarded to the Public Health Institute. This project was partially supported by award number P30CA014089 from the National Cancer Institute. MCS received support from award U54CA233465 from the National Cancer Institute. MCS, JF and SS received support from grants R01CA238087 and R01CA24893 from the National Cancer Institute. MCS, JSM and HJL received support from U2CCA252971 from the National Cancer Institute. The ideas and opinions expressed herein are those of the authors and do not necessarily reflect the opinions of the State of California, Department of Public Health, the National Cancer Institute, and the CDC or their Contractors and Subcontractors.

## CONFLICT OF INTEREST STATEMENT

The authors declare no potential conflicts of interest.

## Supporting information


Table S1.

## Data Availability

The data underlying this article are available in California Cancer Registry Website, at https://www.ccrcal.org/retrieve‐data/. The datasets were derived from sources in the public domain from the California Cancer Registry.
