# Supplementary material for: Disparities among Black and Hispanic colorectal cancer patients: Findings from the California Cancer Registry
Source: Cancer Med. 2023 Nov 1;12(22):20976–88. doi: 10.1002/cam4.6653 (PMC10709728; doi:10.1002/cam4.6653)
Supplement: Supplementary file 1 — Table S1. [file CAM4-12-20976-s001.docx]

| **Supplementary Table 1.** Multivariate logistic regression model of CRC laterality in NHW, NHB, and H/L patients (by nativity) in California with reported tumor location^†^ (1995-2020). | | | | |
| --- | --- | --- | --- | --- |
|  | Right Colon | Left Colon |  |  |
| *(all % are calculated among*  *those with available data only)* | N = 133,246 | N = 90,482 | OR (95% CI) | p-value |
| Population |  |  |  |  |
| NHW | 98798 (74.8%) | 64470 (72.2%) | 1^ref^ |  |
| NHB | 12113 (9.2%) | 7727 (8.7%) | 0.85 (0.83-0.88) | <0.001 |
| USB H/L | 10193 (7.7%) | 8482 (9.5%) | 1.06 (1.03-1.10) | <0.001 |
| NUSB H/L | 10911 (8.3%) | 8574 (9.6%) | 1.05 (1.02-1.08) | 0.001 |
| Missing | 1231 | 1229 |  |  |
| Age at diagnosis |  |  |  |  |
| < 62 | 28023 (21.0%) | 31817 (35.2%) | 1^ref^ |  |
| 62-76 | 50497 (37.9%) | 34657 (38.3%) | 0.61 (0.60-0.62) | <0.001 |
| > 76 | 54726 (41.1%) | 24008 (26.5%) | 0.40 (0.39-0.41) | <0.001 |
| Sex |  |  |  |  |
| Male | 62716 (47.1%) | 49680 (54.9%) | 1^ref^ |  |
| Female | 70513 (52.9%) | 40785 (45.1%) | 0.77 (0.76-0.79) | <0.001 |
| Missing | 17 | 17 |  |  |
| Marital Status |  |  |  |  |
| Single | 17784 (13.3%) | 14218 (15.7%) | 1^ref^ |  |
| Married | 67891 (51.0%) | 48637 (53.8%) | 0.96 (0.94-0.99) | 0.002 |
| Separated/Divorced/Widow | 43135 (32.4%) | 23251 (25.7%) | 0.95 (0.93-0.98) | 0.002 |
| Unknown | 4436 (3.3%) | 4376 (4.8%) | 1.30 (1.26-1.33) | <0.001 |
| CRC, Colorectal Cancer Risk; OR, Odds Ratio; HR, Hazard Rate Ratio; NHW, Non-Hispanic Whites; NHB, Non-Hispanic Blacks; USB H/L, US born Hispanic/Latino/x/a; NUSB H/L, Non-US born Hispanic/Latino/x/a.  ^†^Participants with not otherwise specified tumors in the colon did not contribute to this model (n = 15,104; 6.32% of all colon tumors). | | | | |

| **Supplementary Table 2.** Demographic and clinical characteristics of NHW, NHB, and H/L patients in California who did not receive treatment (1995-2020). | | | | | |
| --- | --- | --- | --- | --- | --- |
|  | NHW | NHB | USB H/Ls | NUSB H/Ls | Heterogeneity |
| *(all % are calculated among*  *those with available data only)* | N = 23674 | N = 3505 | N = 2621 | N = 3364 | p-value |
| Year of diagnosis |  |  |  |  | <0.001 |
| 1995 – 2004 | 8767 (37.0%) | 1188 (33.9%) | 597 (22.8%) | 799 (23.8%) |  |
| 2005 – 2014 | 8967 (37.9%) | 1430 (40.8%) | 1053 (40.2%) | 1375 (40.9%) |  |
| 2015 onwards | 5940 (25.1%) | 887 (25.3%) | 971 (37.0%) | 1190 (35.4%) |  |
| Age at diagnosis |  |  |  |  | <0.001 |
| < 62 | 3401 (14.4%) | 791 (22.6%) | 797 (30.4%) | 889 (26.4%) |  |
| >= 62 and <= 76 | 6360 (26.9%) | 1223 (34.9%) | 886 (33.8%) | 1057 (31.4%) |  |
| > 76 | 13913 (58.8%) | 1491 (42.5%) | 938 (35.8%) | 1418 (42.2%) |  |
| Sex |  |  |  |  | <0.001 |
| Male | 11462 (48.4%) | 1680 (47.9%) | 1553 (59.3%) | 1739 (51.7%) |  |
| Female | 12209 (51.6%) | 1825 (52.1%) | 1067 (40.7%) | 1625 (48.3%) |  |
| Missing | 3 |  | 1 |  |  |
| nSES |  |  |  |  | <0.001 |
| Low nSES | 7670 (32.4%) | 2109 (60.2%) | 1478 (56.4%) | 2194 (65.2%) |  |
| Middle nSES | 5609 (23.7%) | 642 (18.3%) | 541 (20.6%) | 563 (16.7%) |  |
| High nSES | 10395 (43.9%) | 754 (21.5%) | 602 (23.0%) | 607 (18.0%) |  |
| insurance |  |  |  |  | <0.001 |
| Not Insured | 345 (1.5%) | 87 (2.5%) | 55 (2.1%) | 160 (4.8%) |  |
| Managed Care | 6682 (28.2%) | 1005 (28.7%) | 832 (31.7%) | 822 (24.4%) |  |
| Medicaid | 1045 (4.4%) | 336 (9.6%) | 283 (10.8%) | 578 (17.2%) |  |
| Medicare | 11238 (47.5%) | 1509 (43.1%) | 989 (37.7%) | 1283 (38.1%) |  |
| Other | 673 (2.8%) | 155 (4.4%) | 100 (3.8%) | 65 (1.9%) |  |
| Unknown | 3691 (15.6%) | 413 (11.8%) | 362 (13.8%) | 456 (13.6%) |  |
| Marital Status |  |  |  |  | <0.001 |
| Single | 3410 (14.4%) | 966 (27.6%) | 558 (21.3%) | 642 (19.1%) |  |
| Married | 8189 (34.6%) | 913 (26.0%) | 960 (36.6%) | 1371 (40.8%) |  |
| Sep/Divorced/Widowed | 9513 (40.2%) | 1297 (37.0%) | 796 (30.4%) | 1004 (29.8%) |  |
| Unknown | 2562 (10.8%) | 329 (9.4%) | 307 (11.7%) | 347 (10.3%) |  |
| Tumor location |  |  |  |  | <0.001 |
| colon | 16578 (70.5%) | 2559 (73.2%) | 1682 (64.7%) | 2153 (64.7%) |  |
| rectum | 6942 (29.5%) | 935 (26.8%) | 918 (35.3%) | 1177 (35.3%) |  |
| Missing | 154 | 11 | 21 | 34 |  |
| Tumor laterality |  |  |  |  | <0.001 |
| Right colon | 7408 (31.5%) | 1196 (34.2%) | 671 (25.8%) | 931 (28.0%) |  |
| Left colon | 4921 (20.9%) | 781 (22.4%) | 561 (21.6%) | 696 (20.9%) |  |
| Colon NOS | 4249 (18.1%) | 582 (16.7%) | 450 (17.3%) | 526 (15.8%) |  |
| Rectum | 6942 (29.5%) | 935 (26.8%) | 918 (35.3%) | 1177 (35.3%) |  |
| Missing | 154 | 11 | 21 | 34 |  |
| AJCC Stage | |  |  |  | <0.001 |
| Stage I | 2389 (19.5%) | 359 (18.2%) | 272 (19.2%) | 289 (16.8%) |  |
| Stage II | 921 (7.5%) | 100 (5.1%) | 95 (6.7%) | 125 (7.3%) |  |
| Stage III | 359 (2.9%) | 50 (2.5%) | 33 (2.3%) | 60 (3.5%) |  |
| Stage IV | 8589 (70.1%) | 1460 (74.1%) | 1014 (71.7%) | 1246 (72.4%) |  |
| Missing | 11416 | 1536 | 1207 | 1644 |  |
| NHW, Non-Hispanic Whites; NHB, Non-Hispanic Blacks; USB H/L, US born Hispanic/Latino/x/a; NUSB H/L, non-US born Hispanic/Latino/x/a; nSES, Neighborhood-socioeconomic status; NOS, Not otherwise specified. | | | | | |

| **Supplementary Table 3.** Multivariate CRC-specific survival analyses for CRC cases in California (1995-2020) stratified by stage at diagnosis. | | | | | | | | |
| --- | --- | --- | --- | --- | --- | --- | --- | --- |
|  | Stage I | | Stage II | | Stage III | | Stage IV | |
|  | HR^†^  (95% CI) | p-value | HR^†^  (95% CI) | p-value | HR^†^  (95% CI) | p-value | HR^†^  (95% CI) | p-value |
| Population |  |  |  |  |  |  |  |  |
| NHW | 1^ref^ |  | 1^ref^ |  | 1^ref^ |  | 1^ref^ |  |
| NHB | 1.30  (1.20-1.42) | <0.001 | 1.26  (1.18-1.34) | <0.001 | 1.13  (1.08-1.18) | <0.001 | 1.04  (1.01-1.08) | 0.020 |
| USB H/Ls | 1.05  (0.96-1.14) | 0.312 | 1.11  (1.05-1.18) | 0.001 | 1.01  (0.96-1.06) | 0.693 | 0.96  (0.93-0.99) | 0.021 |
| NUSB H/Ls | 0.90  (0.82-0.99) | 0.025 | 0.92  (0.86-0.98) | 0.008 | 0.91  (0.87-0.96) | <0.001 | 0.82  (0.79-0.85) | <0.001 |
| CRC, Colorectal Cancer; HR, Hazard Rate; NHW, Non-Hispanic Whites; NHB, Non-Hispanic Blacks; USB H/L, US born Hispanic/Latino/x/a; NUSB H/L, non-US born Hispanic/Latino/x/a.  ^†^Adjusted for age, sex, neighborhood socioeconomic status, marital status, tumor localization, and treatment status. | | | | | | | | |

| **Supplementary Table 4.** Sociodemographic and clinical characteristics^§^ of NHW, NHB, and non-imputed H/L CRC patients in California (1995-2020). | | | | | |
| --- | --- | --- | --- | --- | --- |
|  | NHW | NHB | USB H/Ls | NUSB H/Ls | Heterogeneity |
|  | N = 248,238 | N = 28,433 | N = 30,345 | N = 32,402 | p-value |
| Year of diagnosis |  |  |  |  | <0.001 |
| 1995 – 2004 | 108158 (43.6%) | 10736 (37.8%) | 5747 (34.4%) | 5713 (26.9%) |  |
| 2005 – 2014 | 93033 (37.5%) | 11850 (41.7%) | 7015 (42.0%) | 9271 (43.6%) |  |
| 2015 onwards | 47047 (19.0%) | 5847 (20.6%) | 3943 (23.6%) | 6281 (29.5%) |  |
| p-value ^†^ |  | <0.001 | <0.001 | <0.001 |  |
| p-value ^‡^ |  |  |  | <0.001 |  |
| Age at diagnosis |  |  |  |  | <0.001 |
| < 62 | 66020 (26.6%) | 10636 (37.4%) | 6905 (41.3%) | 9879 (46.5%) |  |
| 62-76 | 92735 (37.4%) | 11279 (39.7%) | 6229 (37.3%) | 7274 (34.2%) |  |
| > 76 | 89483 (36.0%) | 6518 (22.9%) | 3571 (21.4%) | 4112 (19.3%) |  |
| p-value ^†^ |  | <0.001 | <0.001 | <0.001 |  |
| p-value ^‡^ |  |  |  | <0.001 |  |
| Sex |  |  |  |  |  |
| Females | 128502 (51.8%) | 14237 (50.1%) | 9111 (54.6%) | 11276 (53.0%) |  |
| Males | 119697 (48.2%) | 14193 (49.9%) | 7588 (45.4%) | 9981 (47.0%) |  |
| p-value ^†^ |  | <0.001 | <0.001 | <0.001 |  |
| p-value ^‡^ |  |  |  | 0.003 |  |
| nSES |  |  |  |  | <0.001 |
| Low nSES | 67805 (27.3%) | 15699 (55.2%) | 9033 (54.1%) | 13541 (63.7%) |  |
| Middle nSES | 56014 (22.6%) | 5696 (20.0%) | 3588 (21.5%) | 3631 (17.1%) |  |
| High nSES | 124419 (50.1%) | 7038 (24.8%) | 4084 (24.4%) | 4093 (19.2%) |  |
| p-value ^†^ |  | <0.001 | <0.001 | <0.001 |  |
| p-value ^‡^ |  |  |  | <0.001 |  |
| Insurance Status |  |  |  |  | <0.001 |
| Not Insured | 2400 (1.0%) | 564 (2.0%) | 297 (1.8%) | 1043 (4.9%) |  |
| Managed Care | 109168 (44.0%) | 12233 (43.0%) | 7509 (45.0%) | 7438 (35.0%) |  |
| Medicaid | 9320 (3.8%) | 2748 (9.7%) | 1689 (10.1%) | 4474 (21.0%) |  |
| Medicare | 105637 (42.6%) | 9870 (34.7%) | 5515 (33.0%) | 6519 (30.7%) |  |
| Other | 7194 (2.9%) | 1438 (5.1%) | 838 (5.0%) | 588 (2.8%) |  |
| Unknown | 14519 (5.8%) | 1580 (5.6%) | 857 (5.1%) | 1203 (5.7%) |  |
| p-value ^†^ |  | <0.001 | <0.001 | <0.001 |  |
| p-value ^‡^ |  |  |  | <0.001 |  |
| Marital Status |  |  |  |  | <0.001 |
| Single | 32596 (13.1%) | 7349 (25.8%) | 3150 (18.9%) | 3771 (17.7%) |  |
| Married | 131643 (53.0%) | 10892 (38.3%) | 8644 (51.7%) | 12205 (57.4%) |  |
| Separated/Divorced/Widowed | 74180 (29.9%) | 8669 (30.5%) | 4490 (26.9%) | 4575 (21.5%) |  |
| Unknown | 9819 (4.0%) | 1523 (5.4%) | 421 (2.5%) | 714 (3.4%) |  |
| p-value ^†^ |  | <0.001 | <0.001 | <0.001 |  |
| p-value ^‡^ |  |  |  | <0.001 |  |
| Religion |  |  |  |  | <0.001 |
| Roman catholic | 37967 (15.3%) | 1540 (5.4%) | 8730 (52.3%) | 13276 (62.4%) |  |
| None/agnostic/atheist | 17919 (7.2%) | 1280 (4.5%) | 640 (3.8%) | 611 (2.9%) |  |
| Christian not catholic | 80953 (32.6%) | 14194 (49.9%) | 3133 (18.8%) | 2746 (12.9%) |  |
| Jewish | 8274 (3.3%) | 49 (0.2%) | 44 (0.3%) | 75 (0.4%) |  |
| Other western | 261 (0.1%) | 30 (0.1%) | 6 (<1%) | 9 (<1%) |  |
| Eastern | 1355 (0.6%) | 163 (0.6%) | 20 (0.1%) | 13 (0.1%) |  |
| Unknown | 101509 (40.9%) | 11177 (39.3%) | 4132 (24.7%) | 4535 (21.3%) |  |
| p-value ^†^ |  | <0.001 | <0.001 | <0.001 |  |
| p-value ^‡^ |  |  |  | <0.001 |  |
| Tumor location |  |  |  |  | <0.001 |
| colon | 174243 (71.5%) | 21200 (75.8%) | 10933 (66.9%) | 13524 (65.0%) |  |
| rectum | 69387 (28.5%) | 6778 (24.2%) | 5419 (33.1%) | 7270 (35.0%) |  |
| p-value ^†^ |  | <0.001 | <0.001 | <0.001 |  |
| p-value ^‡^ |  |  |  | <0.001 |  |
| AJCC Stage |  |  |  |  | <0.001 |
| Stage I | 55106 (26.5%) | 5454 (23.9%) | 3242 (23.2%) | 3628 (20.5%) |  |
| Stage II | 58802 (28.2%) | 5588 (24.5%) | 3656 (26.2%) | 4773 (27.0%) |  |
| Stage III | 52273 (25.1%) | 5826 (25.6%) | 3760 (27.0%) | 4983 (28.2%) |  |
| Stage IV | 42045 (20.2%) | 5914 (26.0%) | 3291 (23.6%) | 4297 (24.3%) |  |
| p-value ^†^ |  | <0.001 | <0.001 | <0.001 |  |
| p-value ^‡^ |  |  |  | <0.001 |  |
| Treatment reception |  |  |  |  | <0.001 |
| No treatment | 23674 (9.7%) | 3505 (12.5%) | 1490 (9.1%) | 2010 (9.6%) |  |
| Treatment received | 220659 (90.3%) | 24458 (87.5%) | 14900 (90.9%) | 18861 (90.4%) |  |
| p-value ^†^ |  | <0.001 | 0.012 | 0.783 |  |
| p-value ^‡^ |  |  |  | 0.076 |  |
| Time from diagnosis to treatment |  |  |  |  | <0.001 |
| 0-3 months | 196552 (89.8%) | 21474 (88.4%) | 13056 (88.2%) | 16135 (86.4%) |  |
| 3+ months | 22301 (10.2%) | 2824 (11.6%) | 1742 (11.8%) | 2546 (13.6%) |  |
| p-value ^†^ |  | <0.001 | <0.001 | <0.001 |  |
| p-value ^‡^ |  |  |  | <0.001 |  |
| CRC, Colorectal Cancer; NHW, Non-Hispanic Whites; NHB, Non-Hispanic Blacks; USB H/L, US-born Hispanic/Latino/x/a; NUSB H/L, non-US born Hispanic/Latino/x/a; nSES, Neighborhood-socioeconomic status.  ^†^Versus NHW; ^‡^USB H/L versus NUSB H/L patients; ^§^Only demographic and clinical characteristics included in multivariate analyses are shown for brevity. | | | | | |

| **Supplementary Table 5.** Multivariable logistic regression models for determinants of tumor localization, receipt of treatment and time between diagnosis and treatment in California CRC patients without imputed nativity (1995-2020). | | | | | | | | | |
| --- | --- | --- | --- | --- | --- | --- | --- | --- | --- |
|  | **Tumor localization** | |  | **Receipt of Treatment** | |  | **Time to treatment** | |  |
|  | Colon | Rectum |  | No Treatment | Treatment received |  | 0-3 months | 3+ months |  |
|  | N = 136,357 | N = 57,833 |  | N = 19,551 | N = 174,323 |  | N = 153,734 | N = 19,166 |  |
|  | Odds Ratio (95% CI) | | p-value | Odds Ratio (95% CI) | | p-value | Odds Ratio (95% CI) | | p-value |
| Population |  |  |  |  |  |  |  |  |  |
| NHW | 1^ref^ | |  | 1^ref^ | |  | 1^ref^ | |  |
| NHB | 0.71 (0.69-0.74) | | <0.001 | 0.71 (0.66-0.78) | | <0.001 | 1.15 (1.08-1.22) | | <0.001 |
| USB H/L | 1.07 (1.04-1.11) | | <0.001 | 0.87 (0.80-0.95) | | 0.003 | 1.13 (1.06-1.19) | | <0.001 |
| NUSB H/L | 1.13 (1.10-1.17) | | <0.001 | 0.87 (0.80-0.95) | | 0.001 | 1.29 (1.23-1.36) | | <0.001 |
| Age at diagnosis |  |  |  |  |  |  |  |  |  |
| < 62 | 1^ref^ | |  | 1^ref^ | |  | 1^ref^ | |  |
| 62-76 | 0.64 (0.63-0.66) | | <0.001 | 0.50 (0.46-0.53) | | <0.001 | 1.10 (1.05-1.15) | | <0.001 |
| > 76 | 0.42 (0.41-0.44) | | <0.001 | 0.18 (0.16-0.19) | | <0.001 | 1.10 (1.04-1.15) | | <0.001 |
| Sex |  | |  |  | |  |  | |  |
| Male | 1^ref^ | |  | 1^ref^ | |  | 1^ref^ | |  |
| Female | 0.79 (0.78-0.80) | | <0.001 | 1.06 (1.01-1.11) | | 0.023 | 0.94 (0.91-0.97) | | <0.001 |
| Marital Status |  | |  |  | |  |  | |  |
| Single | 1^ref^ | |  | 1^ref^ | |  | 1^ref^ | |  |
| Married | 0.96 (0.94-0.99) | | 0.015 | 1.69 (1.58-1.81) | | <0.001 | 0.92 (0.87-0.96) | | 0.001 |
| Separated/Divorced/Widow | 0.98 (0.95-1.02) | | 0.324 | 1.10 (1.03-1.18) | | 0.006 | 0.96 (0.91-1.01) | | 0.109 |
| Unknown | 1.17 (1.10-1.24) | | <0.001 | 0.85 (0.74-0.97) | | 0.018 | 1.16 (1.04-1.30) | | 0.007 |
| nSES |  | |  |  | |  |  | |  |
| Low nSES | - | |  | 1^ref^ | | <0.001^†^ | 1^ref^ | | <0.003^†^ |
| Middle nSES | - | |  | 1.13 (1.06-1.20) | | <0.001 | 1.00 (0.96-1.04) | | 0.949 |
| High nSES | - | |  | 1.33 (1.27-1.41) | | <0.001 | 0.95 (0.91-0.99) | | 0.011 |
| Insurance Status |  | |  |  | |  |  | |  |
| Not Insured | - | |  | 1^ref^ | |  | 1^ref^ | |  |
| Managed Care | - | |  | 3.26 (2.82-3.75) | | <0.001 | 1.00 (0.88-1.14) | | 0.947 |
| Medicaid | - | |  | 1.88 (1.61-2.19) | | <0.001 | 1.22 (1.06-1.40) | | 0.005 |
| Medicare | - | |  | 2.79 (2.41-3.22) | | <0.001 | 1.02 (0.90-1.17) | | 0.724 |
| Other | - | |  | 2.02 (1.70-2.39) | | <0.001 | 1.20 (1.03-1.39) | | 0.019 |
| Unknown | - | |  | 1.41 (1.19-1.66) | | <0.001 | 0.94 (0.81-1.11) | | 0.477 |
| Religion |  | |  |  | |  |  | |  |
| Roman Catholic | - | |  | 1^ref^ | |  | - | |  |
| None/Agnostic/  Atheist | - | |  | 0.81 (0.72-0.90) | | <0.000 | - | |  |
| Christian non-Catholic | - | |  | 0.99 (0.93-1.07) | | 0.872 | - | |  |
| Jewish | - | |  | 1.20 (1.03-1.40) | | 0.018 | - | |  |
| Other Western | - | |  | 0.96 (0.45-2.04) | | 0.920 | - | |  |
| Eastern | - | |  | 0.94 (0.67-1.31) | | 0.699 | - | |  |
| Unknown | - | |  | 0.65 (0.61-0.70) | | <0.000 | - | |  |
| AJCC Stage |  | |  |  | |  |  | |  |
| Stage I | - | |  | 1^ref^ | |  | 1^ref^ | |  |
| Stage II | - | |  | 3.31 (3.03-3.62) | | <0.001 | 1.09 (1.04-1.14) | | <0.001 |
| Stage III | - | |  | 6.80 (5.99-7.72) | | <0.001 | 1.06 (1.01-1.11) | | 0.012 |
| Stage IV | - | |  | 0.18 (0.17-0.19) | | <0.001 | 1.20 (1.14-1.26) | | <0.001 |
| Tumor location |  | |  |  | |  |  | |  |
| Colon | - | |  | 1^ref^ | |  | 1^ref^ | |  |
| Rectum | - | |  | 0.85 (0.81-0.90) | | <0.001 | 1.34 (1.29-1.39) | | <0.001 |
| CRC, Colorectal Cancer; NHW, Non-Hispanic Whites; NHB, Non-Hispanic Blacks; USB H/L, US-born Hispanic/Latino/x/a; NUSB H/L, non-US born Hispanic/Latino/x/a; nSES, neighborhood-socioeconomic status.  ^†^P-trend; five-strata nSES variable (with quintiles as cut-off point) was fitted as a continuous variable in independent model. | | | | | | | | | |
